# Supplementary material for: Understanding the Interplay between COX-2 and hTERT in Colorectal Cancer Using a Multi-Omics Analysis
Source: Cancers (Basel). 2019 Oct 11;11(10):1536. doi: 10.3390/cancers11101536 (PMC6827032; doi:10.3390/cancers11101536)
Supplement: Supplementary file 1 [file cancers-11-01536-s001.zip › Supplementary Figures.pdf]

Supplementary Materials

Understanding the Interplay between COX-2 and hTERT in Colorectal Cancer Using a Multi-Omics Analysis

Georgios D. Ayiomamitis, George Notas, Thivi Vasilakaki, Aikaterini Tsavari, Styliani Vederaki, Theodosis Theodosopoulos, Elias Kouroumalis and Apostolos Zaravinos

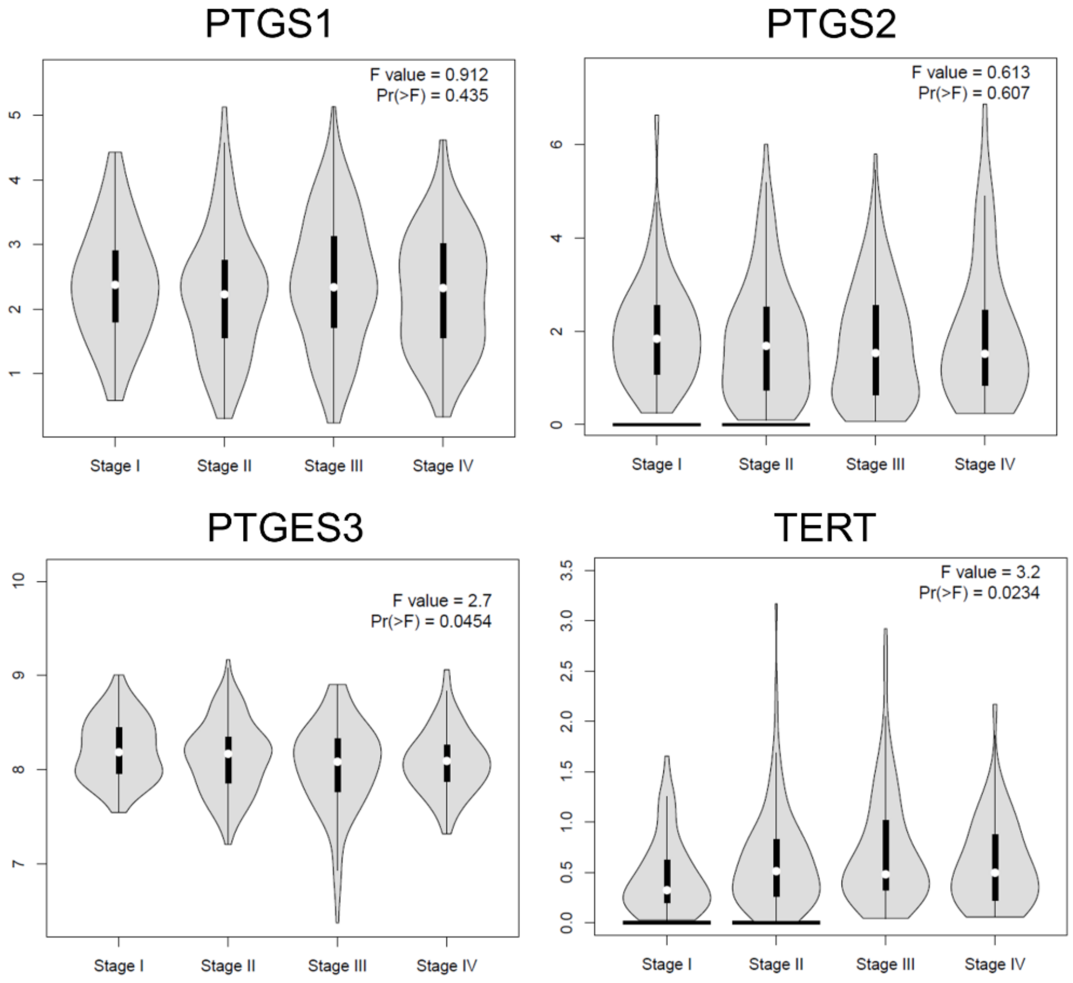

Figure S1. PTGS1 or PTGS2 expression levels did not differ with regard to tumor staging ($p > 0.05$), but PTGES3 expression was lower, and TERT expression was higher in high stage tumors (III and IV), compared to low-stage tumors (I and II).

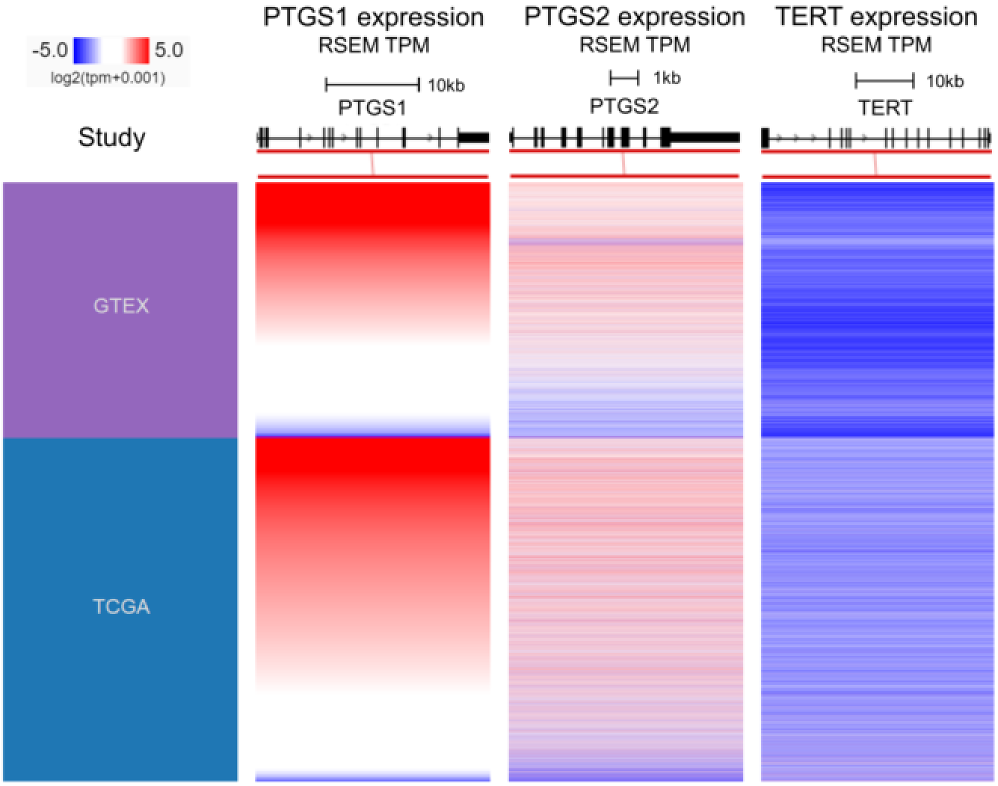

Figure S2. Heatmap depicting the PTGS1, PTGS2 and TERT expression levels (log2(TPM + 0.001)) in the TCGA-COAD and GTEx projects.

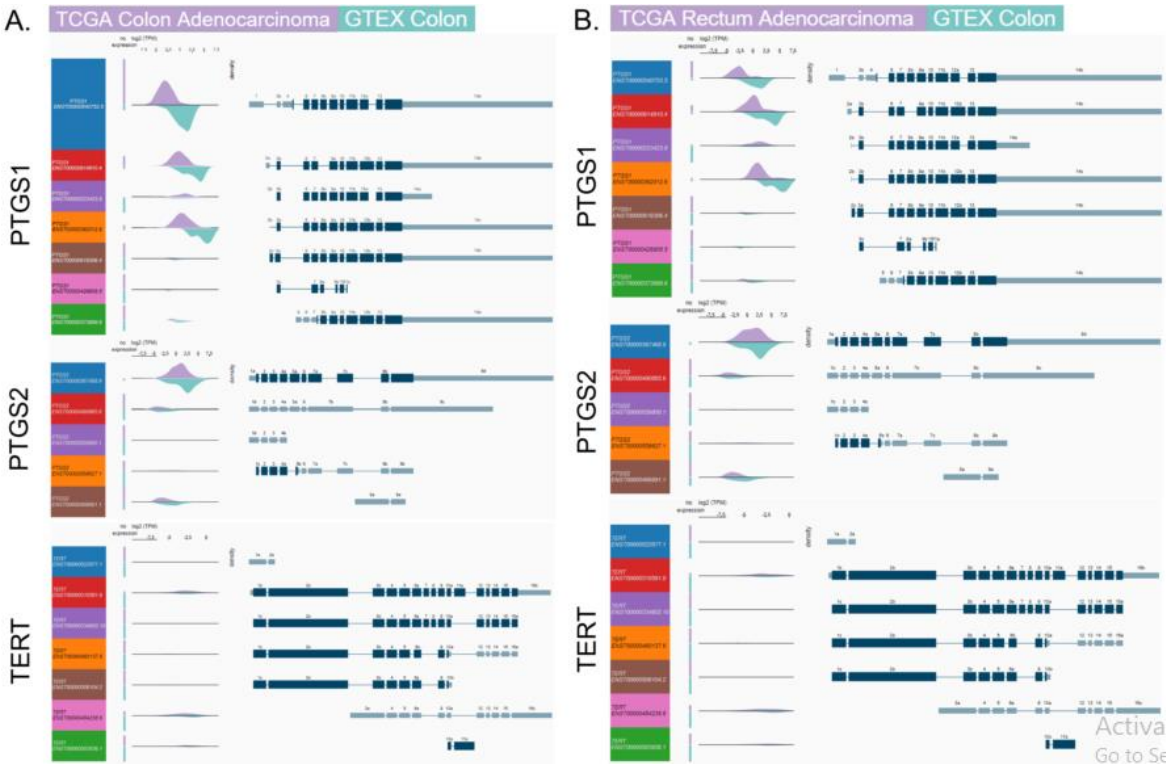

Figure S3. Transcript analysis of PTGS1, PTGS2 and TERT in colorectal cancer (TCGA-Colon/Rectum Adenocarcinoma) vs. the normal colon (GTEx Colon). Expression values of each gene's transcript appear in log_2TPM values.
